# A Case of Nontuberculous Mycobacterial Pulmonary Disease Complicating Massive Pulmonary Embolism

**DOI:** 10.1002/rcr2.70292

**Published:** 2025-07-24

**Authors:** Yasuyuki Hayashi, Akihiko Sokai, Toshiyuki Iwata, Yuki Sakai, Naoaki Yasuda, Takashi Nishimura

**Affiliations:** ^1^ Department of Respiratory Medicine Kyoto Katsura Hospital Kyoto Japan

**Keywords:** bacterial pneumonia, bronchoscopy, chronic thromboembolic pulmonary hypertension, *Mycobacterium avium*, pulmonary embolism

## Abstract

A 39‐year‐old man presented with fever and dyspnoea for 1 week. Imaging suggested bacterial pneumonia with infiltrates in the right lung. However, the symptoms persisted despite antibiotics. Bronchoscopy revealed coagulation necrosis, and enhanced computed tomography identified a large thrombus in the right pulmonary artery, leading to a diagnosis of pulmonary infarction. The patient was treated with direct oral anticoagulants. Two years later, new nodular lesions with cavities were observed in the upper lobe of the right lung. Bronchoscopy revealed a *Mycobacterium avium* infection. We hypothesise that nontuberculous mycobacterial (NTM) pulmonary disease may complicate chronic pulmonary embolism.

## Graphical abstract

We report a rare case of non‐tuberculous mycobacterial disease 2 years after treatment for pulmonary embolism. If a persistent cough or sputum is present after pulmonary embolism, it may be necessary to consider the possibility of NTM pulmonary disease.

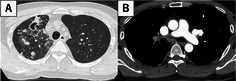

## 1 Introduction

We report a rare case of NTM pulmonary disease 2 years after treatment for pulmonary embolism. Chronic embolism‐induced blood flow disorders may contribute to the development of NTM. If a persistent cough or sputum is present after pulmonary embolism, it may be necessary to consider the possibility of NTM pulmonary disease and perform imaging tests.

## 2 Case Report

**FIGURE 1:**
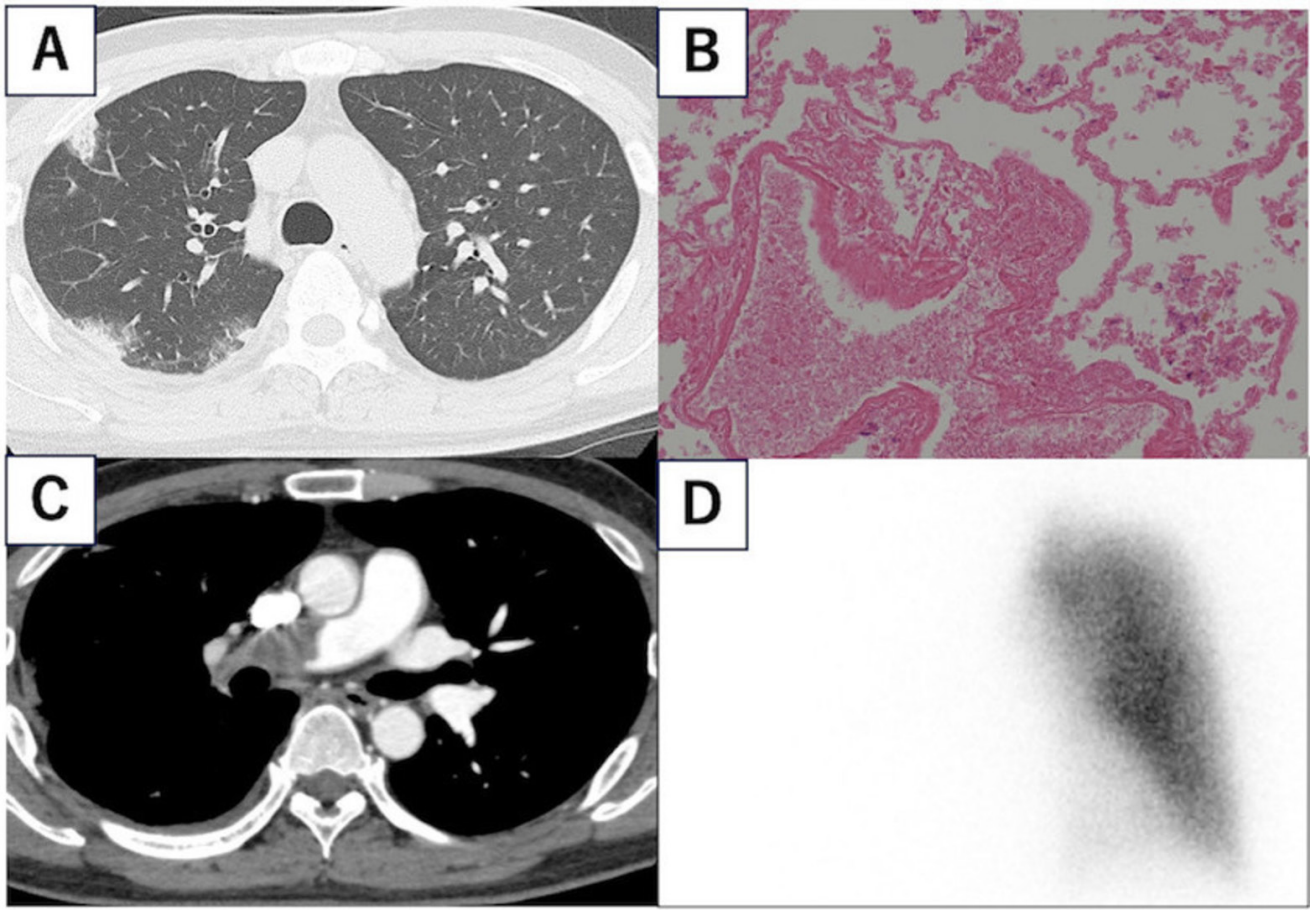
Radiographic and pathological images at first examination. (A) Plain computed tomography (CT) showing scattered infiltrations in the peripheral areas of the right lung lobe. (B) Haematoxylin and eosin staining showing that nuclear staining has disappeared in the blood vessels and alveolar regions, but the structure is preserved. It is an image of coagulation necrosis, suggesting ischaemic changes such as pulmonary infarction. (C) Additional contrast CT showing a massive thrombus in the main trunk of the right pulmonary artery. (D) Lung perfusion scan showing disruption in blood flow to the right lung.

A 39‐year‐old man was referred to our hospital with fever and dyspnoea lasting 1 week. He had a history of *Mycobacterium kansasii* infection at Age 24, which was treated successfully over a year. He had no history of other infectious diseases. He was found to be HIV‐negative, but no other tests for immunodeficiency were performed. On admission, his oxygen saturation level was 94% on room air. Laboratory tests showed a C‐reactive protein (CRP) of 15.3 mg/dL and D‐dimer of 4.4 μg/mL. Computed tomography (CT) showed scattered infiltrates in the peripheral areas of the right lung lobe and right pleural effusion (Figure 1A). Initially diagnosed with bacterial pneumonia, the patient's condition worsened despite antibiotic administration.

Transbronchial biopsy of the infiltrates in the right lung showed coagulative necrosis, suggesting a pulmonary embolism (Figure 1B). Enhanced CT identified a large thrombus in the main trunk of the right pulmonary artery (Figure 1C), and lung perfusion scintigraphy showed no blood flow in the right lung (Figure 1D). The patient was diagnosed with pulmonary infarction due to pulmonary embolism. Treatment with direct oral anticoagulants (DOACs) was initiated instead of thrombolytic therapy and thrombectomy as the patient was hemodynamically stable and did not require oxygen supply.

**FIGURE 2:**
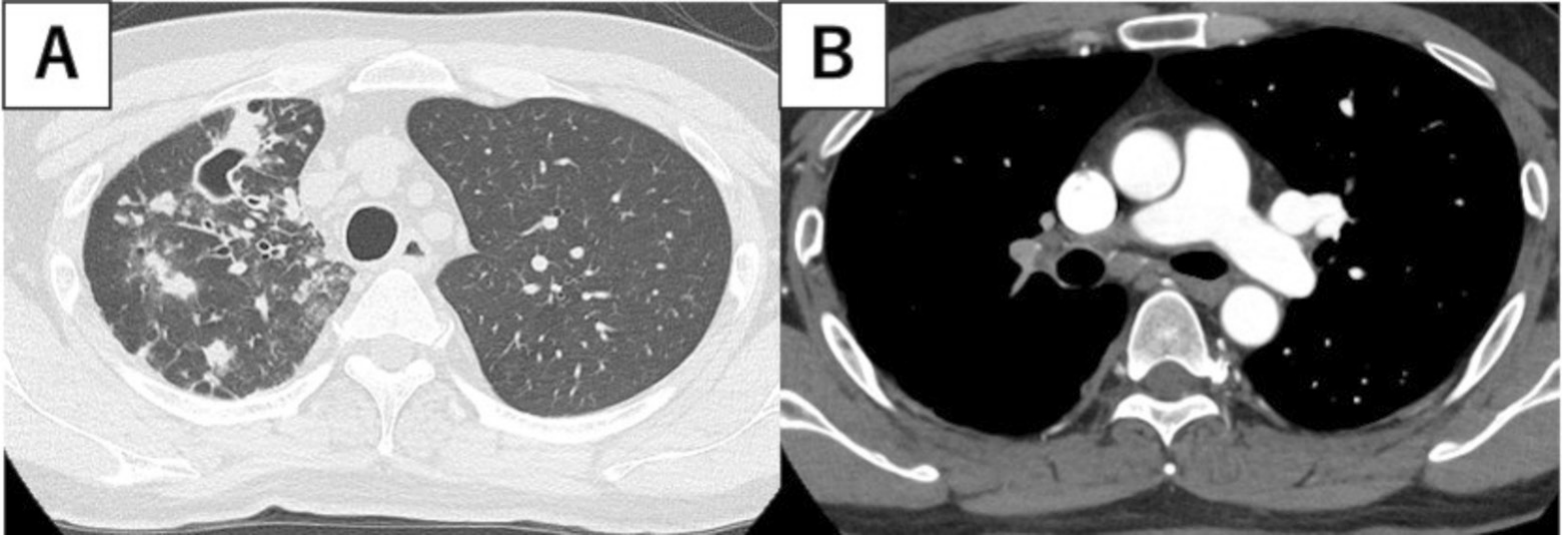
Radiographic images taken 2 years after the first examination. (A) CT showing new nodules with cavities in the upper right lobe. (B) CT showing a residual thrombus in the main trunk of the right pulmonary artery.

After starting treatment for pulmonary infarction, chest CT scans performed every 6 months showed no obvious cavitary lesions or bronchiectasis. However, the chest CT scan performed 2 years later revealed new nodules with cavities in the right upper lobe (Figure 2A) The thrombus remained, with developed collateral blood vessels (Figure 2B). Bronchoscopy identified *Mycobacterium avium* in the bronchoalveolar lavage fluid and tissue culture from the right upper lobe. The diagnostic criteria for NTM pulmonary disease were met based on CT and bronchoscopy findings. Drug susceptibility testing for NTM was not performed due to resource limitations, so susceptibility was unknown. The patient was diagnosed with NTM pulmonary disease, complicating pulmonary infarction, and treated with rifampicin, clarithromycin, and ethambutol. After 2 years of oral treatment, some nodules in the right lung shrank, whereas the cavities persisted. The treatment response was slow. However, there was no worsening of imaging findings or subjective symptoms, so medication was discontinued after 2 years of treatment. Sputum cultures remained negative throughout the treatment period, and there was no radiological deterioration or microbiology to suggest recurrence for more than 2 years after treatment completion.

## 3 Discussion

NTM are environmental organisms capable of causing opportunistic pulmonary disease when bacteria are inhaled from water sources or soil [1]. Host factors, including immunosuppressive conditions and lung structural abnormalities, such as COPD and bronchiectasis, significantly contribute to the risk of NTM pulmonary disease [2]. A retrospective study reported that Chronic Thromboembolic Pulmonary Hypertension (CTEPH) may predispose individuals to NTM pulmonary disease, with a higher incidence in segments of the lung where the pulmonary artery is obstructed compared to healthy segments [3]. It has been suggested that the macrophage activity and interferon gamma may affect NTM susceptibility [4, 5]. In patients with pulmonary embolism, including those with CTEPH, chronic embolism‐induced blood flow disorders may contribute to the development of NTM. Restoration of blood flow by pulmonary endarterectomy (PEA) may enhance immune response and improve treatment outcomes for NTM in these patients [3]. Therefore, NTM pulmonary disease may complicate chronic pulmonary embolism, with interruption of pulmonary blood flow potentially leading to a poor response to treatment.

## Author Contributions

Yasuyuki Hayashi and Akihiko Sokai conceptualised and drafted the initial manuscript. All authors reviewed and edited the manuscript.

## Consent

The authors declare that written informed consent was obtained for the publication of this manuscript and accompanying images using the consent form provided by the Journal.

## Conflicts of Interest

The authors declare no conflicts of interest.

## Data Availability

The data that support the findings of this study are available from the corresponding author upon reasonable request.
